# Computation of Steady-State Probability Distributions in Stochastic Models of Cellular Networks

**DOI:** 10.1371/journal.pcbi.1002209

**Published:** 2011-10-13

**Authors:** Mark Hallen, Bochong Li, Yu Tanouchi, Cheemeng Tan, Mike West, Lingchong You

**Affiliations:** 1 Department of Biomedical Engineering, Duke University, Durham, North Carolina, United States of America; 2 Structural Biology and Biophysics Program, Duke University, Durham, North Carolina, United States of America; 3 Department of Statistical Science, Duke University, Durham, North Carolina, United States of America; 4 Center for Systems Biology, Duke University, Durham, North Carolina, United States of America; 5 Institute for Genome Sciences and Policy, Duke University, Durham, North Carolina, United States of America; University of Illinois at Urbana-Champaign, United States of America

## Abstract

Cellular processes are “noisy”. In each cell, concentrations of molecules are subject to random fluctuations due to the small numbers of these molecules and to environmental perturbations. While noise varies with time, it is often measured at steady state, for example by flow cytometry. When interrogating aspects of a cellular network by such steady-state measurements of network components, a key need is to develop efficient methods to simulate and compute these distributions. We describe innovations in stochastic modeling coupled with approaches to this computational challenge: first, an approach to modeling intrinsic noise via solution of the chemical master equation, and second, a convolution technique to account for contributions of extrinsic noise. We show how these techniques can be combined in a streamlined procedure for evaluation of different sources of variability in a biochemical network. Evaluation and illustrations are given in analysis of two well-characterized synthetic gene circuits, as well as a signaling network underlying the mammalian cell cycle entry.

## Author Summary

Variability from one cell to another is a pronounced and universal trend in living organisms; much of this variability is related to varying concentrations of proteins and other chemical species across the cells. Understanding this variability is necessary if we are to fully understand cellular functions, particularly the ways in which cells differ from each other and in which cells with the same origin behave in different ways (e.g. in human development and cancer). When using a chemical model for some aspect of cellular function, one needs to consider two sources of variability: intrinsic variability, which results from the reactions proceeding as in the model but naturally varying because of the finite number of molecules in the cells and their random behavior; and extrinsic variability, which results from other kinds of variation not accounted for in the specific, deterministic model. We present new methods to model and compute both kinds of variability, to facilitate the study of cellular variability as a whole. Our methods provide advantages in speed, accuracy, and scope of mechanisms modeled, and we apply them to experimental data, demonstrating the nature of intrinsic and extrinsic noise in those systems.

## Introduction

Cellular processes are “noisy”. In each cell, concentrations of molecules (e.g., mRNAs, proteins) are subject to random fluctuations (noise) due to the small numbers of these molecules and to environmental perturbations [1], [2]. Cellular noise impacts on information transmission involved in cell signaling dynamics [3]–[5], while cells may take advantage of such variability in adapting to changing environments or for cell-fate decisions [6]–[15]. Improved understanding of how noise influences and is modulated by cellular processes will greatly benefit from efficient, streamlined computational tools to quantify noise, and to use noise to probe properties of the underlying regulatory networks [16]–[19]. To date, stochastic modeling of gene expression has typically relied on forward simulations of time courses, for example via Gillespie algorithms [20], [21] or numerical solution of stochastic differential equations (SDEs) [4], [22], [23].

Flow cytometry and fluorescence microscopy currently allow for access to increasingly rich data on approximately steady-state distributions of gene expression. These distributions arise biologically when a set of reactions proceeds much faster than environmental changes, and observing such data provides a step towards understanding some aspects of the underlying cellular network. To assess how such data can be informative, we need to compute or simulate aspects of the steady-state distribution. Forward simulation can be time-consuming, and new approaches are needed. Approaches such as umbrella sampling [24] and coupling-from-the-past [25] have been introduced, but the sampling biases of the former and substantial computational expenses of the latter leave areas for improvement.

Mechanistic modeling of noise is complicated by its diverse sources, which have been classified as intrinsic or extrinsic [26], [27]. Intrinsic noise results from the stochasticity of chemical kinetics when the numbers of interacting molecules are sufficiently small; it can be described by the chemical master equation (CME). In essence, intrinsic noise represents deviation of known reactions with known rates from their results as predicted by classical chemical kinetics [28]. In contrast, extrinsic noise results from other reactions and from fluctuations in rate constants, and it is often the dominant source of variability in a system [26], [29]. Extrinsic noise may result from any process not represented in the network model itself.

A direct route to model intrinsic noise is to calculate steady-state solutions to the CME, often by using an approximation. An analytical solution based on a continuous master equation describing protein production in bursts has been formulated by Friedman and colleagues [30], while Fourier and colleagues [31] present analytical solutions for several other networks. Walczak and colleagues [32] investigate another solution approach based on using an eigenbasis from a simpler system to solve the massive linear equation resulting from setting the CME to steady-state. The approximation here lies in the difference between each system's eigenbases, and its suitability for a specific system needs to be determined on an *ad hoc* basis. More general methods have been investigated as well. The Hartree approximation [33] assumes probabilistic independence of molecule numbers for each species; this approximation greatly reduces the dimensionality of the system, but tends to break down seriously in multimodal systems, unless the joint distribution has a mode at each combination of the one-dimensional distributions' modes (this is frequently not the case). Cao and colleagues [34], [35] investigate accurate though computationally costly numerical solution methods for the CME, such as efficient exhaustive enumeration of microstates. Munksy and Khammash [36], focusing on the application of methods for solving the master equation, investigate the necessary data for obtaining reaction parameters in a system dominated by this type of noise.

A related approach is to calculate an “energy landscape” for a network. Ao [37] assumes an SDE model and derives a potential that yields the probability distribution as its Boltzmann distribution. Wang and colleagues [38] also use an SDE model and then construct a potential landscape based on a Hodge decomposition of the flux vector in the system. Both approaches are useful for a wide range of SDEs, including the chemical Langevin equation. However, they are thus subject to the inaccuracies of that equation—most importantly, the inaccuracy at low molecule numbers—and may also lack computational tractability for complex systems. Qian and Beard [39], in constructing potential landscapes for non-equilibrium systems based on chemical potentials, provide an approximation for the probability distribution that follows the Hartree approximation.

In contrast to intrinsic noise, extrinsic noise lacks a unique modeling framework and is often determined by empirical inference of distributions from data. One approach that accounts for some of these effects is to perturb the rate constants while modeling intrinsic fluctuations using a Gillespie algorithm-type simulation strategy [40]. This approach may also produce extrinsic fluctuations that could be produced by other sources, such as other reactions and measurement noise. However, direct steady-state calculations can instead pool together results from many extrinsically perturbed distributions, thus preventing the need to perform calculations for many parameter sets and many time points. Analytical inclusion of extrinsic noise is also possible, and indeed the use of exponentially distributed burst sizes in modeling protein production in [30] amounts to this. In addition, extrinsic noise can be accounted for by addition of random noise to molecule numbers in each time step of a timecourse simulation based on a stochastic differential equation [4], [23]. Recent single-molecule fluorescent measurements have allowed experimental determination of molecule number distributions in *Escherichia coli*, thus measuring both intrinsic and extrinsic noise [41].

Despite these progresses, a major challenge lies in the lack of well-defined computational framework for thorough, systematic evaluation of these methods with experimental data. As a step to address these issues, we have developed an integrated framework for modeling steady-state distributions in the context of both intrinsic and extrinsic noise sources. As an illustration, we have applied these methods to the analysis of two well-characterized bistable switches and evaluated the methods against experimental data. Furthermore, we also demonstrated the applicability of these methods to a more complex signaling network, the Myc/Rb/E2F network, which underlies the control of mammalian cell cycle entry.

## Results

### Overview of the Computational Framework

**Figure 1:**
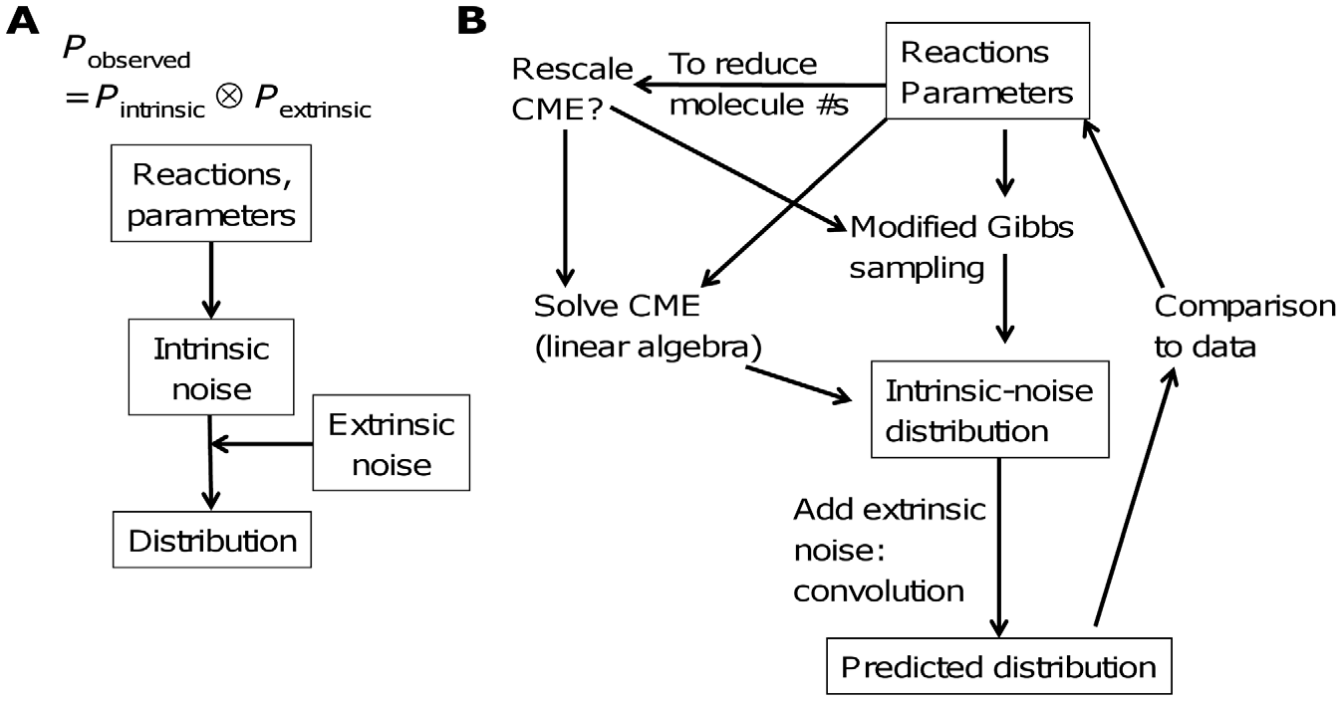
A framework for combining intrinsic and extrinsic noise. (A) Distribution prediction starts with predicting distributions based on intrinsic noise only and then adds in extrinsic noise. (B) A schematic for analysis of molecule number distributions in biochemical networks. Predicted distributions based on a model can be compared to experimental data, and information about parameters can be inferred.

In general, the observed distribution of molecular counts (*P*_observed_) can be treated as the combination of an intrinsic component (*P*_intrinsic_) and an extrinsic component (*P*_extrinsic_) (Figure 1A). The intrinsic component is uniquely determined by the reaction mechanisms and the corresponding rate constants. Our approach (Figure 1B) takes a list of species, reactions with known rate information, and known extrinsic noise parameters, and at the first step calculates the steady-state distribution based on the chemical master equation. This first step accounts for intrinsic noise implicitly and can be done analytically for systems with a sufficiently small number of states. When the CME is too complicated to solve analytically, it can be solved numerically to generate the steady-state distributions, up to the size and dimension limits imposed by computational capabilities. The CME is of the form **MP** = 0, where **M** is a matrix and **P** is the steady-state probability vector.

With many reacting species, as the matrix size may imply prohibitive computation cost, we can rescale the CME or sample approximately from the solution. For scaling, the dimension of the space of distributions is reduced by approximating the CME in terms of directional derivatives and then re-sampling. The scaled CME is then solved by linear algebra. Even with scaling, however, the matrix computations needed to solve the CME become prohibitive when more than a few species are present or when the distribution is complex. We address these limitations by developing a modified Gibbs sampling (MGS) method to generate the steady-state solution to the CME. Gibbs sampling provides a set of samples from a distribution by sampling one dimension of the distribution (in this case, the molecule number for a given species) at a time, using the conditional distribution for that species given the current molecule numbers of all the other species. In our MGS method, detailed balance is assumed for different sets of reactions at each iteration, generating approximate conditional distributions from which exact sampling is possible. The MGS method scales much more favorably than the direct CME solution with increased numbers of species. Its scaling property is similar to that of ordinary Gibbs sampling. Importantly, it overcomes some caveats associated with alternative approximations, especially in multimodal systems. In particular, it avoids the restrictions on the distribution space caused by the Hartree approximation. Also, it overcomes the difficulties in sampling multiple local minima that occur with the standard Gibbs sampler.

The second step of our approach is to model extrinsic noise by convolution. Typically, representing extrinsic noise as perturbations to rate parameters [40] can present significant difficulties in their application to experimental data. Sampling from a parameter sample space would lead to high computational cost because of the need to redo calculations for many different parameter sets. Methods based on adding noise at each time step similarly bring the cost of calculation at many unnecessary points in time. To this end, we have developed a convolution approach to represent extrinsic noise by averaging many effects, which allows more direct application to experimental data. It is well suited to combining analysis of the modes by a deterministic model, allowing rapid and accurate estimation of reaction parameters, with estimation of further noise parameters based on the observed distribution.

### Derivations

#### Chemical master equation (CME)

The CME describes temporal evolution of the probability of a given state, as represented by a set of molecule numbers for all species in the system:
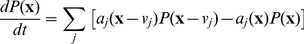
(1)where *P*(**x**) denotes the probability of the system being in state **x** as a function of time, *t*; *v_j_* denotes the change in **x** resulting from reaction *j*, and *a_j_*(**x**) denotes the probability of reaction *j* per unit time given that the system is in state **x** (i.e. the rate in molecular units).

At steady state, the time derivative is 0 for all *P*(**x**). This results in a linear system of equations for the probabilities of states: the reaction rates define a matrix, and the null vector of this matrix, normalized so its elements sum to 1, is the vector of probabilities of states.

#### Synthesis and degradation of a single molecule

Consider a simple system consisting of *n* molecules, with synthesis rate *a_s_(n)* and degradation rate *a_d_(n)*. The corresponding CME and its steady-state solution are:
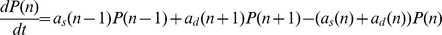


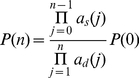
(2)
*P*(0) is chosen such that the sum of all probabilities is 1. For the constitutive expression of a single protein, *a_s_*(*n*) = *k_s_*, *a_d_*(*n*) = *k_d_n*, where *k_s_* is the synthesis rate and *k_d_* is the decay rate constant. Here *P*(*n*) follows a Poisson distribution with mean *k_s_*/*k_d_*:
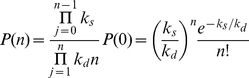
(3)


This distribution describes the variability resulting from intrinsic noise. To account for extrinsic noise, one approach is to draw the parameters from their own probability distributions (assuming that the stochasticity from other cellular processes is manifested in fluctuations in the rate constants), which need to be empirically determined:
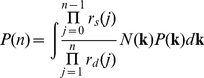
(4)where **k** is the vector of parameters, *P*(**k**) is its probability density function, *N*(**k**) is a normalization constant, *r_s_* is the synthesis rate constant, *r_d_* is the degradation rate constant, and the integral is over all values of *k* with nonzero probability.

In the simple example above, the distribution is determined by the parameter *y* = *k_s_*/*k_d_*, so drawing *y* from a gamma distribution with parameters *α* and θ,
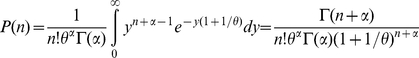
(5)Here, we chose the gamma distribution because positive real values of **k** are needed and the gamma distribution draws values from this set and allows the integral to be performed analytically. Choosing separate distributions for *k_s_* and *k_d_* is not necessary here because only the corresponding distribution of *y* would affect the final distribution of *n*.

#### Scaling of the CME

Excessive matrix size can present a major challenge for solving the CME, both as a result of high dimensionality and of high individual molecule numbers (though not high enough for classical reaction rate equations to be used). The size of this equation may be reduced by approximating it in terms of derivatives and then resampling it,
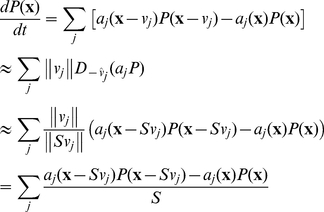
(6)where *S* (≥1) is a scale factor (*S* = 1 denotes no scaling) and *D* is a directional derivative, allowing general use of this formalism regardless of what reactions are in the system; the sums in the first and third lines are finite-difference approximations of the directional derivative in the second line.

Several scales may be used for the same problem: the unscaled equation can be used for small molecule numbers while less dense sampling may be used for larger ones where the linear approximation applies better. If computer memory is a greater barrier than computation time in solving the steady-state CME, it may be appropriate to choose *S* as small as possible without running out of memory, since the maximum molecule numbers that need to be included in an analysis are essentially independent of scaling. A discrete, rescaled CME avoids the numerical error that would likely result from a continuous approximation. Also, it reduces exactly to the unscaled master equation when *S* = 1. This is useful for direct comparison of systems of different sizes. It is also useful for systems where the molecule numbers for some but not all species are large enough to require scaling.

The synthesis/degradation system is a useful test case of the scaling method. Combining Eqs 2 and 6, the scaled master equation for this system is
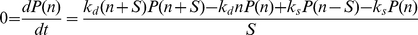
(7)thus completely determining *P*(*n*) up to normalization. Provided sufficiently small variation between each *P*(*n*) and *P*(*n−S*), the normalization
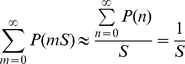
(8)may be applied.

Eq. 7 gives *P*(*n*)>*P*(*n*−*S*) if *n<k_s_/k_d_* and *P*(*n*)<*P*(*n*−*S*) if *n>k_s_/k_d_*; thus the distribution peaks at *n = k_s_/k_d_*, as is the case without scaling. Furthermore, for *S* = 1, it reduces to the ordinary CME, as is the case in general for Eq. 6.

#### Modeling intrinsic noise via Gibbs sampling

High dimensionality can make accurate numerical solution of CME intractable, even with scaling. One way to effectively reduce the dimensionality is to use Gibbs sampling [42], [43]. Each step in Gibbs sampling requires constructing only a 1-D distribution. To sample, one cycles through the *d* different species, sampling a value for each molecule number *x_i_* in turn from the conditional distribution *P(x_i_|x_1_,…,x_i−1_,x_i+1_,…x_d_)* of the molecule number being sampled given the other current molecule numbers. This yields a sample from the entire distribution, (*x_1_,…,x_d_*) at the conclusion of each cycle. Gibbs sampling for the steady-state solution of the CME can be performed by assuming detailed balance for synthesis and degradation of a given species; that is:
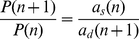
(9)where *n* is the molecule number of the species; *a_s_* and *a_d_* are its synthesis and degradation rates.

This equation can be modified to account for reactions with different stoichiometries by simply replacing *n* and *n+1* with the states interconverted by the other reactions (e.g. one can replace *n+1* with *n+2* if molecules are synthesized and degraded two at a time).

**Figure 2:**
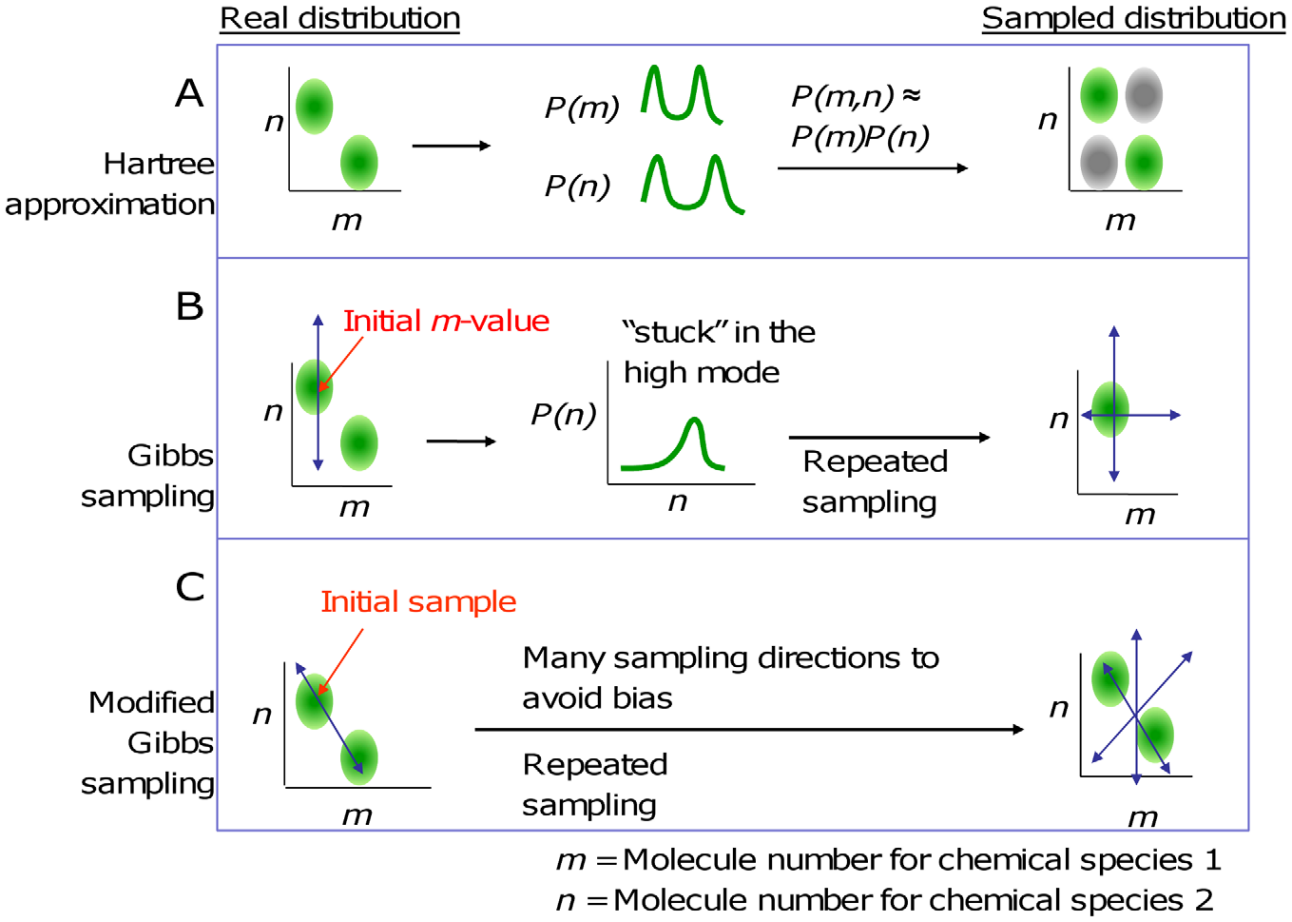
A modified Gibbs sampling method in comparison to previous methods. (A) The Hartree approximation can distort the joint distribution for multimodal distributions by generating false peaks. (B) Gibbs sampling method: sampling of each molecule number is based on current values of the other molecule numbers rather than on mean values, to avoid this distortion. This method can result in samples being “stuck” in one peak of the probability distribution. The blue arrows in (B) and (C) indicate sampling directions, which are used sequentially. (C) A modified Gibbs sampling method based on coordinate changes can avoid the sampling bias.

The sampling algorithm starts with an arbitrary initial value for all but one of the molecule numbers, calculates the distribution of the remaining molecule number by assuming detailed balance (Eq. 9) with the other numbers fixed. This sample is then fixed when other species are being sampled. Each complete sampling cycle yields a new sampled state. This strategy is similar to the mean-field method often used with the Hartree approximation (Figure 2A). However, because it samples from a distribution for one species that is correct for the current sample's (rather than the mean's) molecule numbers of the other species, it avoids the key pitfalls in the Hartree approximation when applied to multimodal distributions (Figure 2B).

The basic Gibbs sampling has its own caveat when applied to multimodal distributions: Once a sample is drawn from one peak of such a distribution, it is unlikely to cross over to other peaks, unless the peaks overlap in at least one dimension or if there is sufficient probability density outside the peaks for the samples to migrate between peaks. If the total probabilities of the two peaks are known, one can solve this problem by sampling separately from each peak, at the expense of additional burn-in (samples that must be discarded because they are biased by the initial values).

To overcome this caveat, our modified Gibbs sampling (MGS) method changes coordinates between sampling steps (Figure 2C). An accurate Gibbs sampling scheme needs to accurately draw a sample *x′* given that the previous sample, *x*, is from the correct distribution; in other words it must have *P*(*x′*) = *P*(*x*). This is achieved when *P*(*x′*|*x*) = *P*(*x*|*x′*), because this condition leads to *P*(*x′*; *x*)/*P*(*x*) = *P*(*x′*; *x*)/*P*(*x′*). Changing coordinates between steps by random rotation of the coordinate system satisfies this condition. In 2-D, for example, new axes can be created with slope *m* (where tan^−1^(*m*) is uniformly distributed between −90° and 90°) and −1/*m* (note that tan^−1^(−1/*m*) and tan(*m*) have the same distribution); the axes pass through the previously sampled point. Since the purpose of constructing the axes is to sample from points that lie along them, integer lattice points in one coordinate system must match integer lattice points in the original coordinate system, so that each state can be represented by integer coordinates in the new system. This can be achieved by rounding. In our example, we can let *x* and *y* be the new coordinates and *u* and *v* the old ones, and then let the *x*-axis pass through
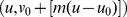
(10)for each value of *u* and the *y*-axis through
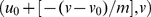
(11)for each value of *v*, where the coordinates are given in the *u*−*v* system, brackets denote rounding to the nearest integer, and (*u_0_*,*v_0_*) is the previously sampled point. This technique is applied to the toggle switch below.

The basic Gibbs sampling algorithm may also fail in monomodal systems when the peak contains a fraction significantly less than 1 of the probability density and the remaining density is widely distributed over a much larger space than the peak occupies. For example, if the peak is at the origin in a high-dimensional state space, the sampler will remain at the origin for a very large amount of time once it is there, and likewise will take an extremely large amount of time to find the origin once it is removed from it in several dimensions (because it will take many steps for all the dimensions to reach 0 or sufficiently close to 0 randomly). Switching to a hyperspherical coordinate system will remove this problem: the sampler may move from the origin to any other state in one step.

Higher accuracy in high-dimensional systems can be achieved by sampling from a 2-D rather than a 1-D distribution. Gibbs sampling can use scaling to curtail excessive molecule numbers too. Overall, MGS provides an efficient way to sample from the probability distribution associated with a steady-state CME even when many species are involved. While the detailed balance approximation introduces some error, this error is mitigated relative to that found in the Hartree approximation. The change of coordinates mitigates the error further because detailed balance for different reactions is chosen for each sample.

Since each iteration of MGS is identical to an iteration of regular Gibbs sampling in the coordinate system that applies at the moment, the computational time of the algorithm is essentially the same as for regular Gibbs sampling. The cost per iteration is slightly larger because of the coordinate-selection step, but this is very fast compared to the actual sampling operation. Furthermore, convergence to the equilibrium distribution occurs on the same timescale that regular Gibbs sampling would exhibit if the latter could accurately sample the distribution. That is, the algorithms should converge similarly as long as the standard Gibbs sampling performs well.

Despite this strong system-dependence, rigorous methods have been developed to evaluate convergence: see for example the convergence criteria of Zellner and Min [44] as well as the convergence analysis of Frigessi and colleagues [45] and the burn-in analysis of Jones and Hobert [46]. In a *d*-dimensional system, each sample requires *d* one-dimensional sampling procedures; the time required for each sampling will tend to be O(*d* ^1/2^) because, even measuring in the worst dimension, the width of the region of state space needing to be sampled scales roughly as *d* ^1/2^. We note that these estimates are somewhat ambiguous because the computational cost of the algorithm varies from system to system. Since each system has a given dimensionality, a generic comparison of computational cost for different dimensionalities must be rather approximate. In particular, the convergence time may depend more on the geometry of the distribution than on the number of species or other generic information.

Furthermore, the principle behind the standard Gibbs algorithm that allows for accurate sampling from the distribution still applies. Consider a sample **x** = (*x*_1_, *x*_2_, …) from the desired distribution. One desires to provide another sample **y** = (*y*_1_, *y*_2_,…), also from the desired distribution. This is achieved because
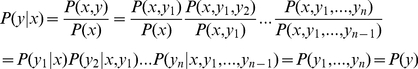
(12)


Sampling from the molecule number distribution rather than calculating it explicitly is advantageous when a model has many dimensions but only a few are needed in the output distribution. Since the observed convergence rate of the distribution will depend primarily on the dimensionality of the observed joint distribution rather than on the overall dimensionality of the model, this results in roughly O(*d* ^1.5^ *m*) computational cost for MGS, where the model has *d* dimensions and the output has *m*. This is much better than the approximately O(*d* ^3^) cost for solving the CME by linear algebra, e.g. by using singular value decomposition, especially if *m*≪*d*. Note that for practical applications, even with large numbers of species, one will often need the distribution of a single species or the joint distribution of a few, because of the impracticality of experimentally monitoring all the species in a complex system.

#### Convolution representation of extrinsic noise

Mechanistic methods of representing extrinsic noise, such as modeling perturbations in each parameter, present significant difficulties in their application to experimental data. Sampling from a parameter sample space leads to high computational cost because of the need to redo calculations for many different parameter sets; methods based on adding noise at each time step, similarly, bring the cost of calculation at many unnecessary points in time. Also, the variation in many parameters will result in an excessive number of degrees of freedom in the extrinsic noise, and still there are likely to be sources of noise unaccounted for because they are not easily represented as perturbations in reaction propensities.

To address this limitation, we present a convolution representation of extrinsic noise based on averaging together many effects and modeling the distribution of extrinsic noise with a set of parameters that can be characterized experimentally. In particular, we represent the total distribution as a weighted integral of shifted intrinsic-noise-only distributions, i.e. the convolution of the intrinsic-noise-only distribution with a distribution of shifts. The rationale for this approach is that most perturbations will induce some shift in the distribution. For example, a reaction not accounted for by the model, or an increase in one of the rate parameters, may affect the concentration of some species. They may also change the shape of the distributions for these species, but this is a less important effect and furthermore will result in a distribution that is a linear combination of shifted versions of the original, intrinsic-noise-only distribution (with the main shifted distribution dominating this combination). The distribution of shifts must have the same dimensionality as the output distribution—note that this is often much less than the dimensionality of the model, thus greatly reducing the number of parameters needed relative to the parameter-variation representation, since there are almost always more reactions (and thus reaction rate parameters) than species in a biochemical network model.

The following derivation shows how convolution can result from parameter-variation descriptions of extrinsic noise. Let *P*(**x**;**k**) denote the probability of a state vector given a parameter set **k**. Based on parameter perturbations as the source of extrinsic noise, the total probability *P*(**x**) of a given state vector is
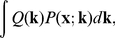
(13)where the integral is over all possible parameter sets and *Q*(**k**) denotes the probability distribution of them, centered around a value **k_0_** (i.e. if trying to measure a single set of parameters for the system, one would be trying to measure **k_0_**). We can let
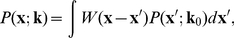
(14)where *W* is a distribution of shifts due to a perturbation in rate parameters (which may depend on **k**). *W* will tend to be a narrow distribution, similar to δ(**x′**−**y**) for some state **y**: changing **k_0_** to **k** will produce primarily a shift in state. Thus
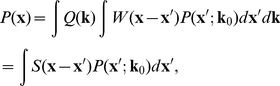
(15)where *S*(**x**−**x′**) is a distribution of shifts, which is convolved with *P*(**x**;**k**) to yield *P*(**x**). *S* may be well approximated by a normal distribution, as it is a sum of many weakly correlated terms (corresponding to the different perturbations). In particular, values of **x′** near the original state vector **x** are likely to have more parameter sets with shift distributions *W* centered near **x′**; also *Q*(**k**) is likely to be higher for **k** nearer to **k_0_**, which in general will correspond to **x′** being nearer to **x**. Note that the strategy used in the above derivation can be applied to perturbations beyond those in the model parameters.

When measuring parameters, it is advantageous to define the extrinsic noise such that it does not induce a systematic shift in the molecule numbers (this merely represents a choice of what base parameter set **k_0_** to use for a parameter distribution *Q*(**k**)); this would correspond to *S*(**x**−**x′**) having zero mean. Also, it might be useful, in a concrete representation of the extrinsic noise distribution, to add a background term to help make up for the approximations in the representation; let this be *B*(**x**). Thus, we may represent *P*(**x**) as
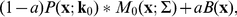
(16)where * denotes convolution, *M_0_* is a zero-mean multivariate normal distribution with the given covariance matrix, and *a* is a small constant.

*M_0_* may have an altered normalization in order to still be a valid probability distribution in the necessary discrete state space (i.e. *M_0_* must still sum to 1 over all states, even though the values are still proportional to what the multivariate normal distribution would ordinarily give). The covariance matrix quantifies the “spreading” of probability density in state space due to extrinsic noise. The background noise term represents the distribution of extrinsic noise far from the values predicted by intrinsic noise. Its associated parameters could be estimated from those sections of a flow cytometry data set only, reducing the number of parameters needed to fit the actual peaks. Fitting of the peaks is facilitated by the fact that their location limits the possible values of the rate parameters, allowing the spread to be used to estimate the extrinsic noise standard deviations.

While convolution can be considered as another form of explicit account for parametric perturbations (with *a priori* specified distributions), its formulation is more general and allows representation of noise sources not well described by perturbations in rate parameters. Many kinds of perturbations can be represented as shifts in the distribution and accounted for by convolution. Because perturbations may induce different shifts in molecule numbers depending on the specific region of state space. For example, a reaction may have a minor effect on molecule numbers when the original molecule numbers are small and a more significant effect when they are large. Therefore, it may be necessary to have the covariance matrix in Eq. 16 depend on **x**, or more generally to have the form of *S* in Eq.15 depend on **x**. For example, different values of the extrinsic noise standard deviation σ can be used for each of the two peaks in a bimodal distribution. In other words, it is preferable to consider Eq. 16 as an empirical equation that has solid theoretic foundation but can be readily used to describe experimental data under diverse conditions.

Either direct summation or (to save time) a discrete Fourier transform is appropriate for performing this convolution if the probability is known in functional form (i.e. if it was obtained by solving the CME exactly or approximately). If only a sample of *n* points **x_1_**,…**x_i_**,…,**x_n_** from the intrinsic-noise-only probability distribution is known, i.e.
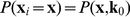
(17)then we can draw *n* new samples **y_1_**,…**y_i_**,…,**y_n_** using
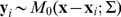
(18)and these obey the convoluted distribution:
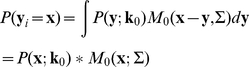
(19)


Once the sample based on the convolution has been drawn in this fashion, if a background noise term in the distribution is desired, then a sample of *na*/(1−*a*) points corresponding to the background term can be drawn to complete the sample based on extrinsic noise. As in any formulation of extrinsic noise, the sources, form, and magnitude of the noise must be determined based on empirical considerations for a given system.

Our convolution approach provides an intuitive relationship between the distribution of extrinsic noise and the final distribution. In principle, it is highly versatile and can account for all sources of extrinsic noise in an empirical manner. As such, determining an appropriate extrinsic–noise distribution will depend on experimental measurements of the overall distribution. Due to the exceedingly complex nature of extrinsic noise in many systems, however, a Gaussian distribution may be the best starting point. On one hand, such a simple distribution avoids overfitting experimental data. On the other, the Gaussian distribution can be thought of modeling extrinsic noise from many weakly correlated sources added together. A significant deviation from model prediction and experimental data would suggest that a large, concerted phenomenon is influencing the extrinsic noise; in that case, it would be appropriate to expand the network model to include this phenomenon explicitly. The convolution approach, as a method for calculating the steady-state distribution, is also useful in that it can model noise from processes at a variety of different timescales—whether these are similar timescales to those of the main network model or not.

### Case Studies

Combining MGS, CME rescaling, and convolution model for extrinsic noise defines an integrated framework for efficient computation of the steady-state distribution of gene expression for a given set of parameters. To illustrate their use, we consider the application of the overall framework to several examples; aspects of these have been mentioned in the previous section.

#### Constitutive expression of a protein (Figure 3A)

**Figure 3:**
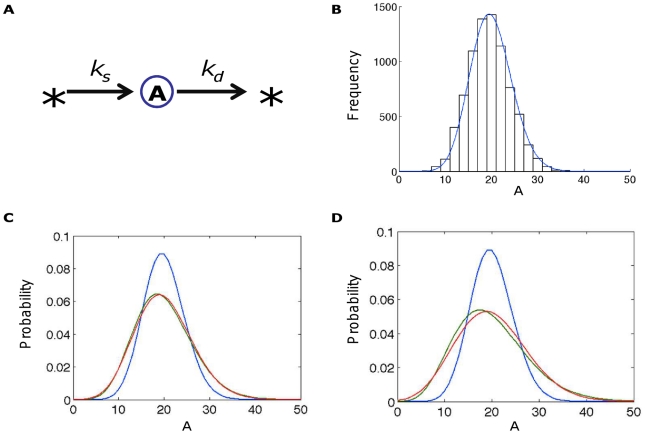
Calculating steady-state distributions for a simple birth-death process. Calculating the steady-state distribution of a (A) simple birth-death process; A is expressed in terms of molecule number for all distributions. (B) Simulated distribution using the Gillespie algorithm (histogram) as compared with the analytical solution (blue line) from the CME, which is a Poisson distribution. (C) Final distributions from this systems with extrinsic noise were generated by taking *k_s_*/*k_d_*∼Γ(20,1) (green in C) or *k_s_*/*k_d_*∼Γ(10,2) (green in D); best-fit distributions based on convolution with a Gaussian (red; standard deviations 4.4 for C and 6.1 for D) and the intrinsic-noise-only distribution (blue) are shown as well. (The shift to lower molecule numbers arising from extrinsic noise in the parameter-distribution representation is equivalent to changing the base parameter set in the convolution representation; the “base” parameter set is less well defined in the parameter-distribution representation).

Here, the intrinsic noise is described exactly by a Poisson distribution (see Eq. 3). As expected, this matches the distribution given by running the Gillespie algorithm for a sufficient length of time (Figure 3B). Integrating over the prior distribution of the parameters allows the analytical addition of extrinsic noise (see Eq. 4). Addition of extrinsic noise results in spreading and, ultimately, loss of the characteristic shape of the distribution (Figure 3C,D). Also, adding extrinsic noise by perturbing parameters or by convolution with a Gaussian produces similar results, as evident from the best-fit convolved distributions to the parameter-perturbed distributions in Figure 3C,D. However, the parameter-perturbation method shifts the peak of the distribution while the convolution method does not. In this sense, the two methods define the “intrinsic-noise-only” state somewhat differently, as the parameter perturbation method considers it to be at the mean value of the parameters while the convolution method (at least when using a Gaussian) maintains the mean of the molecule number distribution, giving a more intuitive description of extrinsic noise. A better match between the techniques could be obtained by matching the distributions of extrinsic noise, as opposed to using a gamma distribution for parameters and a Gaussian for convolution because of the suitability of each of these distributions for their respective methods.

#### A genetic toggle switch

**Figure 4:**
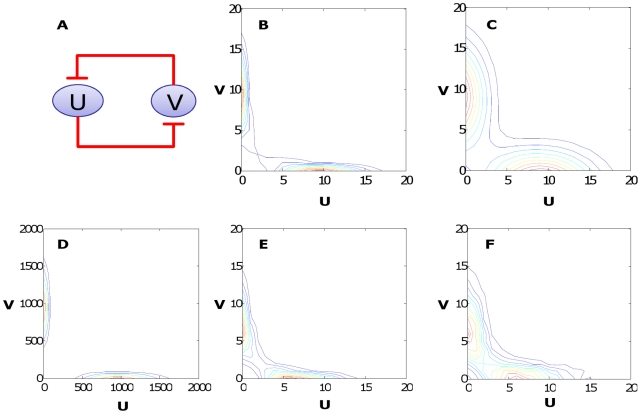
Calculating steady-state distributions for a toggle switch. (A) Circuit diagram. (B) The probability distribution, based only on intrinsic noise, for parameters *K_u_* = *K_v_* = 1, *r_u_* = *r_v_* = 10, β = γ = 2, *d_u_* = *d_v_* = 1. This was calculated by solving the CME directly. U and V are expressed in molecule numbers for each distribution. (C) The same distribution except with extrinsic noise added (σ_1_ = σ_2_ = 2); peak spreading is evident. (D) The intrinsic-noise-only distribution except with *r_u_* = *r_v_* = 1000; calculated using scaling; peak focusing is evident. (E) A sample (10000 points) from this distribution (intrinsic noise only), using modified Gibbs sampling using the same parameters as in (B). (F) Another 10,000-point sample, generated the same way except with extrinsic noise (σ_1_ = σ_2_ = 1).

The toggle switch [47], [48] consists of two proteins that repress each other's expression (Figure 4A). Its reaction kinetics can be described by a highly simplified model consisting of two differential equations (Eqs. 20 and 21, Methods). These equations can then be used to construct the CME model (Eq 22) to describe the corresponding stochastic dynamics (accounting for intrinsic noise only).

The system is bistable given appropriate parameter values, leading to a bimodal distribution by directly solving the CME (Figure 4B). Addition of extrinsic noise by convolution widens the peaks (Figure 4C), while increasing molecule numbers (and therefore, necessarily relying on scaling of the CME to solve it) narrows them (Figure 4D).

With this system, we also compared MGS with the exact solution of the CME. The MGS overall matches direct CME solution very well (compare Figure 4E and 4B); Table 1 shows a quantitative comparison. Using parameter set 1, the probability distribution obtained by directly solving the CME and two approximations based on 10,000 points each from MGS were compared using the sums of squared deviations, which were 0.0095 and 0.011 respectively. For comparison, the two approximations had a sum of squared deviations of 0.0004, and the maximum possible sum of squared deviations is 2. Overall, MGS effectively approximates the true solution of the CME with greatly reduced computational cost.

**Table 1:** Comparison of MGS and direct CME solution for the toggle switch.

Parameter set	Species	Mean (direct)	Mean (MGS)	St. dev. (direct)	St. dev. (MGS)	ON% (direct)	ON% (MGS)
1	U	6.4314	4.0084	5.0680	4.0241	69%	55%
1	V	2.6319	3.0232	4.1081	3.5994		
2	U	3.3446	3.7456	2.5215	2.6332	70%	77%
2	V	1.3271	1.0201	1.8958	1.5593		

Parameter set 1 is: *K_u_* = *K_v_* = 1, *r_u_* = 10, *r_v_* = 9, β = γ = 2, *d_u_* = *d_v_* = 1; parameter set 2 differs in that *r_u_* = *r_v_* = 5 and β = 1.

Addition of extrinsic noise is very quick using the convolution method, since the noise is simply added to the final samples, and the results are similar to those obtained by solving the CME directly (Figure 4F). Note that the effectiveness of Gibbs sampling is in spite of the violation of detailed balance for this system. The appropriateness of the detailed balance approximation may be evaluated by comparing the actual ratios of probabilities for different states to the probabilities assumed by the detailed-balance approximation (Eq. 9). For the parameter set used in Figure 4E, the average relative error of the detailed-balance approximation, weighted by probability, is 0.58 for protein V and 0.25 for protein U.

**Figure 5:**
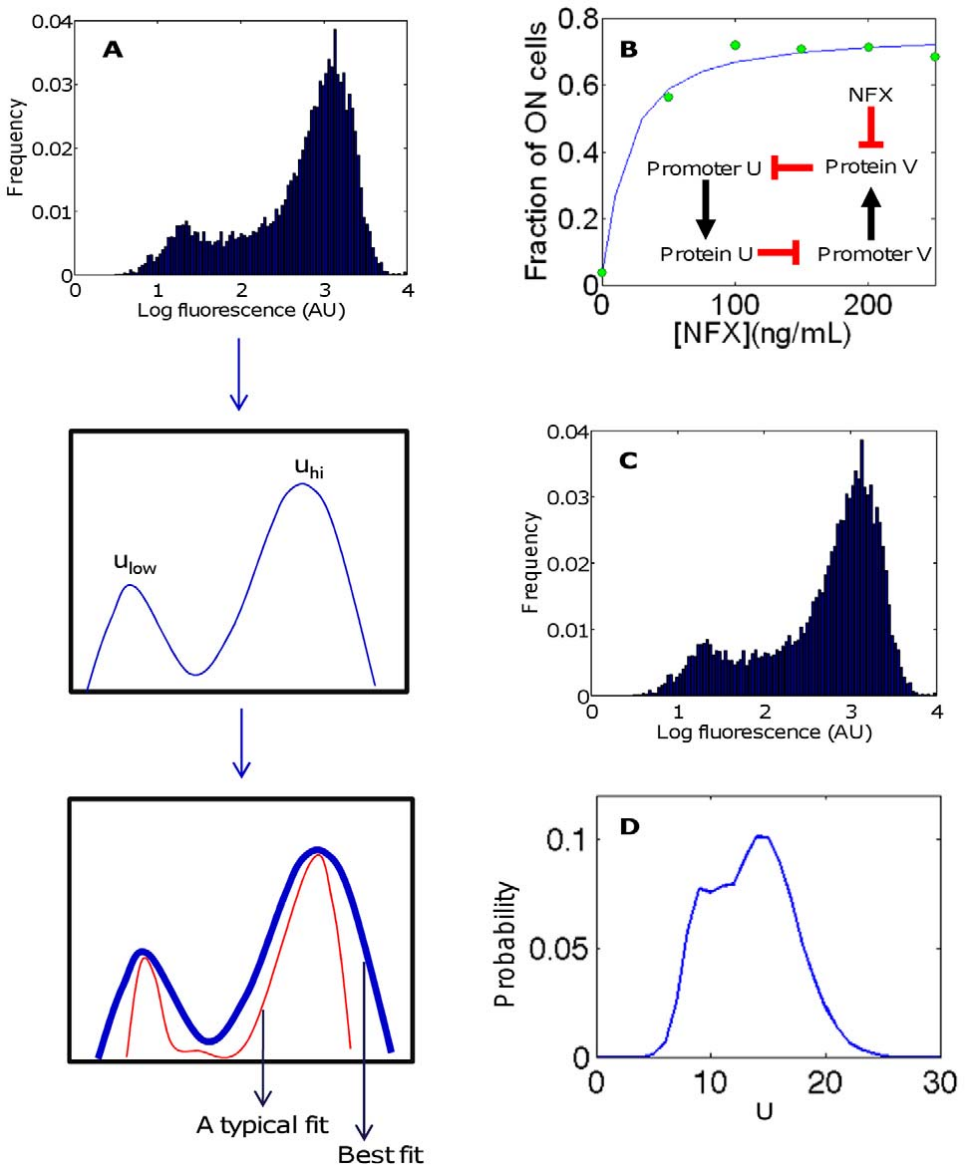
Toggle switch: comparison with experiments. (A) A typical workflow for analyzing experimental data based on the framework for noise calculations presented in this study. Modes of the distribution(s) are determined and used with the deterministic model to estimate some reaction parameters (i.e. intrinsic noise parameters); prior knowledge may also be included in these estimates. Then, using the calculation methods (e.g. convolution for extrinsic noise) presented here, one can obtain a best fit for the full parameter set, which describes both intrinsic and extrinsic noise. (B) Theoretical (blue) and experimental (green) fraction of ON cells as a function of [NFX]. Inset shows the perturbation to the circuit. (C) Experimental distribution of GFP fluorescence in cells with 250 ng/mL NFX. (D) Predicted distribution of U molecule numbers (proportional to fluorescence) with 250 ng/mL NFX. Note that the distribution in (C) is used in (A) to illustrate the general computational procedure.

To illustrate the use of our modeling framework to the analysis of experimental data (Figure 5A), we experimentally measured the switching dynamics using the toggle switch implemented by Kobayashi et al. [48], in response to varying concentrations of the antibiotic norfloxacin (NFX). NFX causes an SOS response, which induces GFP by increasing degradation of its repressor (an additional term is added to the degradation rate constant for the repressor; see Methods for modeling details). The method described above for predicting distributions based on known parameters can be applied to this data, generating reasonable parameter sets for the perturbation. In particular, the model accurately predicted the variation of ON fractions of the population with the concentration of antibiotic (Figure 5B). Parameter sets were obtained by nonlinear fitting. Random searches of parameter space, as well as previous work with similar circuits, were used in the production of initial estimates. In general, the fitting process began with estimation of reaction parameters based on the modes of distributions observed and on previous studies, followed by refinement of these parameters and estimation of others based on nonlinear fitting of the distributions. Notably, the parameter set chosen yields a distribution dominated by unexpectedly low molecule numbers, but an adequate fit was not obtained with higher molecule numbers. The obtained parameter set was *K_u_* = 2.22, *K_v_* = 13.69, *r_u_* = 1306.4, *r_v_* = 35.06, *β* = 5.36, *γ* = 1, *d_u_* = 79.84, *d_v0_* = 0.42, *d_v1_* = 1.61, *k_A_* = 36.70, σ_1_ = 1, and σ_2_ = 1 (see Eqs. 20–23 for parameter definitions; σ_1_ and σ_2_ are standard deviations of shift distributions for proteins U and V respectively). The shapes of the predicted distributions deviated somewhat from the data but were improved by using different levels of extrinsic noise for the two peaks (Figure 5 C,D). The plots show the distributions for 250 ng/mL NFX; the predicted distribution in Figure 5D uses σ = 10^−5^ for the ON peak and σ = 1.4 for the OFF peak, where σ is the standard deviation of the Gaussian distribution describing extrinsic noise (see Eq. 16). The peak is also shifted to account for background fluorescence or synthesis: peaks in the experimental distribution are shifted away from zero.

Results of the fitting indicate that a parameter set of this size is sufficient to describe some properties of the distribution (e.g. dependence of the fraction of ON cells on the circuit induction level). However, good fits of entire distributions, which have many more degrees of freedom, are not possible based on the mechanistic models described and on a simple form for extrinsic noise. The discrepancy likely results from the simplicity of the underlying model that generates the intrinsic variability, or the simplicity of the form of assumed extrinsic noise. In theory, any distribution can be fit to the intrinsic noise model if no constraints are imposed on the extrinsic noise distribution, but such a fit would yield no mechanistic insight without alternative methods to interpret the resulting extrinsic noise term. In principle, however, the computational framework proposed here can be used as an effective approach for comparing different mechanistic models for a given set of data. This aspect will require further in-depth analysis.

#### A growth-modulating positive feedback circuit

**Figure 6:**
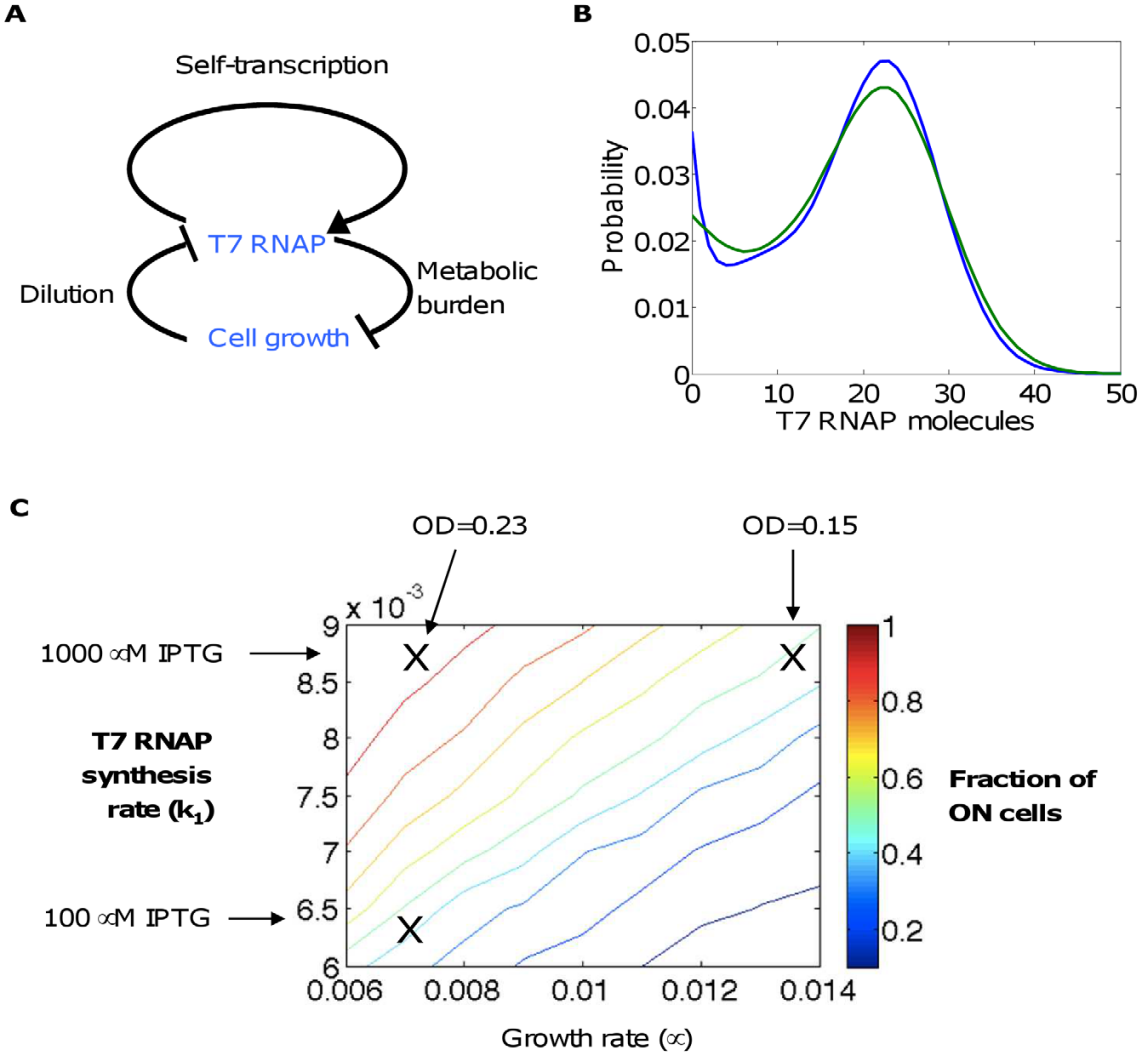
T7 RNAP circuit. (A) T7 RNAP enhances its own transcription. In addition, T7 RNAP expression slows down cell growth, which dilutes T7 RNAP. (B) Probability distribution with *M* = 10, *k*_0_ = 0.001, *k_f_* = 0.01, *k_b_* = 0.1, *k*_1_ = 0.01, *d_x0_* = 0.003, μ = 0.01, and θ = 1; shown with (green) and without (blue) extrinsic noise (σ = 3). (C) Comparison of experiment and modeling on the perturbations to growth and T7 RNAP synthesis rates. Contours denote computed fractions of ON cells, by varying parameters *k*_1_ and μ. X's denote experimental data points with experimental parameters (IPTG concentration and OD) as labeled; the *k*_1_ and μ values for each data point were determined by fitting the fractions of ON cells.

This circuit consists of a T7 RNA polymerase (T7 RNAP) activating its own transcription [49]. It provides a useful example for analyzing a unique mode of regulation of circuit dynamics: in addition to the positive feedback, expression of T7 RNAP slows growth, and growth causes dilution of the T7 RNAP (Figure 6A). As with the toggle switch, the CME model (Eq 27) predicts a bimodal distribution would result for appropriate parameters (Figure 6B,C). This distribution is based on a constant growth rate (i.e. log-phase growth): the bimodality requires constant growth, in which the growth rate and protein synthesis rate are inversely related. In the absence of growth (e.g. during stationary phase), the system would in theory approach a single, non-trivial steady state that would result in a monomodal distribution. In an appropriate time window like exponential growth phase, however, the bimodal distribution can be considered as approximately at steady state (if needed, it can be perpetuated by periodic dilution of the culture). In other words, this reaction can only reach a true steady state if the amount of medium is increasing proportionally to the bacterial growth, allowing perpetual log-phase growth, but a “pseudo-steady-state” corresponding to the limit of slow change in growth rate compared to chemical reactions is possible and interesting. This might, for example, be relevant when there is a large supply of nutrients. These states are the same on a cellular level and are modeled here.

According to this premise, we previously used a Gillespie algorithm to predict how the approximately steady-state distribution would be modulated by inoculum size of the bacteria, which would essentially affect the effective growth rate of the population: the larger the inoculum, the smaller the effective growth rate. The previous modeling predicted that the fraction of ON cells at the final data point would increase with the inoculum size (as controlled by the initial cell density, measured by optical density (OD)) or the circuit induction level (controlled by the concentration of IPTG). This prediction was indeed consistent with experimental measurements.

In this study, we re-evaluated these data using methods presented here. We treated the distributions of the final data point as being at a pseudo-steady state. We varied parameters to match the perturbations in ON (high-T7 RNAP) and OFF (low-T7 RNAP) populations seen in experiments (Figure 6C). As for the toggle switch, initial guesses were obtained with the aid of random searching of parameter space, and nonlinear fitting was used to obtain the final parameter set. The base case of bacterial culture with an initial OD of 0.23 and 1000 µM IPTG exhibited 94% ON cells; this matched a parameter set of *k_0_* = 0.0011, *k_f_* = 0.01, *k_b_* = 0.11, *k*_1_ = 0.0087, *d_x_*_0_ = 0.003, μ = 0.007, and θ = 1 (see Eq. 24, 27). An experiment with the same IPTG level but an initial OD of 0.15, which would result in overall faster growth, exhibited 48% ON cells. We further performed a model fit to determine the decrease in growth rate corresponding to this shift to the OFF population, and the new growth rate was found to be μ = 0.013 (all other parameters were kept the same). Another experiment used OD = 0.23 but provided only 100 µM IPTG, resulting in 49% ON cells. This was modeled as a proportional decrease in *k*_0_ and *k*_1_, which, by fitting, was a 26% decrease in each of these parameters, giving *k*_0_ = 0.0008, *k*_1_ = 0.0065, and other parameters as in the base case. For all numerical analysis, we assumed that the number of promoters to be ten.

Again, here we find that aspects of the experimental data can be readily fitted to the simple model. However, an apparent caveat is that the distributions that we fitted were not genuinely at steady state. Furthermore, similar issues as in the case for the toggle switch also apply, which include the simplicity of the mechanistic model and that in the specific form of the extrinsic noise distribution.

#### Myc/Rb/E2F network

**Figure 7:**
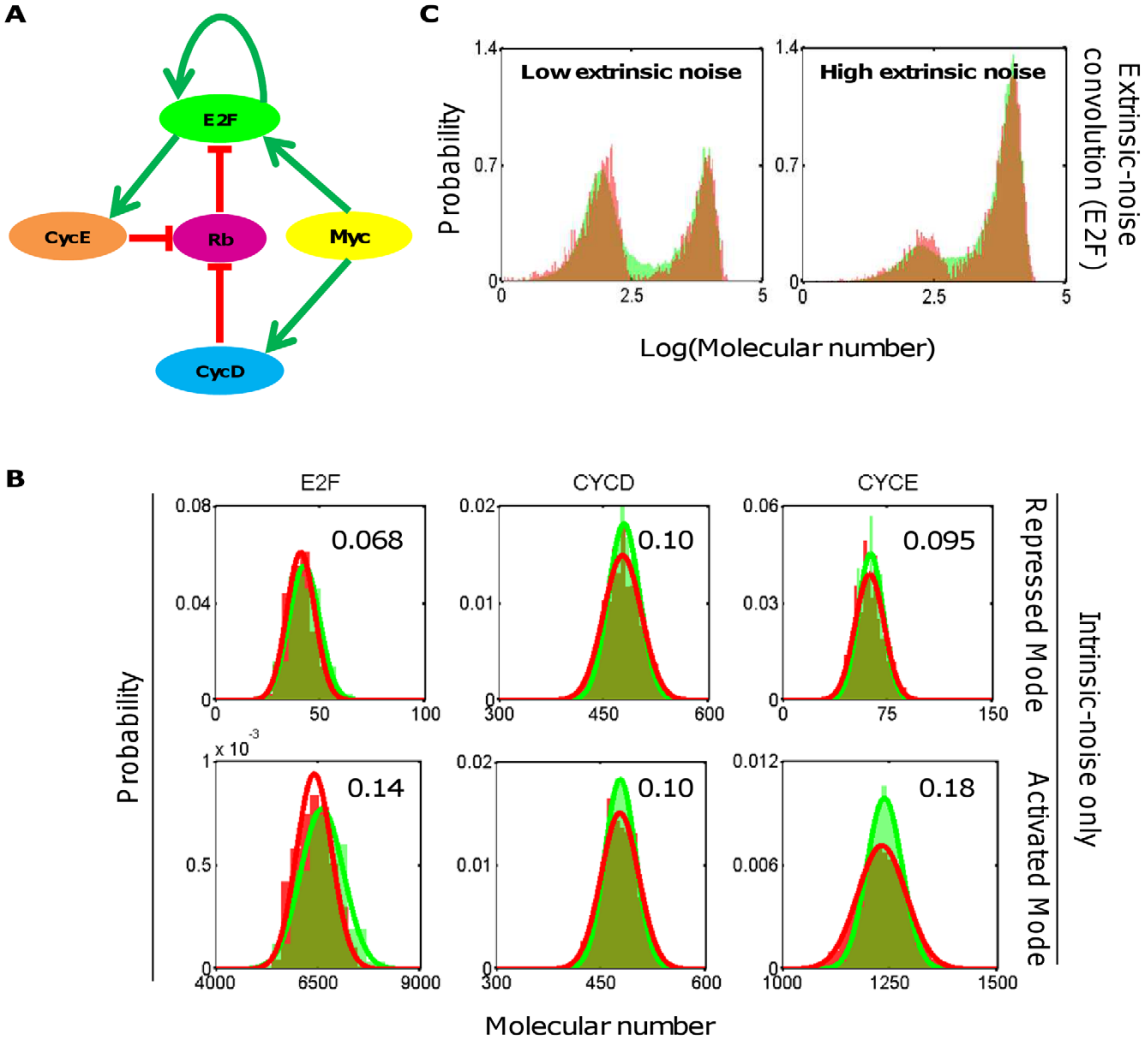
Myc/Rb/E2F network. (A) A network diagram of Myc/Rb/E2F at the G1-S cell cycle checkpoint. (B) Comparison of the MGS predicted probability distribution (red) and the SDEs predicted distribution (green) of selected molecular species of the repressed and the activated state of the network. The solid lines represent Gaussian distributions fitted over all random samples corresponding to each method. (C) Convolution of extrinsic noise and combination of the repressed and the activated modes based on empirical data derived from SDE simulations, with the variance of the shift distribution separately optimized. Shown in red are MGS predicted distributions and green are SDEs predicted distributions of E2F with either low or high level of extrinsic noise as defined in the SDE framework. The SDE model and the associated parameters are described in detail in Lee et al [51].

To illustrate general applicability of our computational framework, we applied it the Myc/Rb/E2F network, which we have analyzed extensively in recent studies (Figure 7A) [50]. This network plays a critical role in regulating cell cycle progression and cell-fate decisions [50]. Here we focus on analyzing the bistable E2F response to serum stimulation [50]. To evaluate the methods described here, we use a well-established stochastic model that we recently developed [51]. The stochastic model consists of a set of stochastic differential equations in the general form of Eq. 28 [51], [52], where extrinsic noise can be introduced as an additive term. When this term is set to 0, the model will generate fluctuations due to intrinsic noise only. These stochastic dynamics can also be fully described by the corresponding CME model (Eq. 29).

For this model, we note that *ad hoc* strategies can be employed to speed up the efficiency of Gibbs sampling. In particular, for the base model parameters, multiple rounds of numerical simulations using the SDE model, with either low or high initial E2F conditions and without the extrinsic noise term, predict a clear bimodal distribution in E2F, with the low mode (obtained with low initial E2F) corresponding to E2F in repressed state and the high mode (obtained with high initial E2F) corresponding to E2F being activated. In fact, the two stable states are so separated that stochastic transition between them by intrinsic noise alone is extremely rare (never observed during SDE simulations once the network settles in either state, data not shown). Therefore, we separately sample the repressed and the activated mode when applying the MGS. The locations of the different modes were estimated using the deterministic model *a priori*, with the same rate parameters.

As shown in Figure 7B, the intrinsic-noise-only distributions of selected species of the Myc/Rb/E2F network (E2F, CYCD, and CYCE) generated by MGS (red) closely resemble those predicted by the SDE model (green). The sums of squared deviations between SDEs-predicted distributions and MGS-predicted distributions are shown besides each plot.

Next, we incorporated an additive extrinsic noise into the SDEs (as described in [51]) to simulate empirical stochastic system output, equivalent to experimental data one would expect. Again, we note that significant extrinsic noise is required for the transition between the repressed and the activated mode. As such, we performed the extrinsic noise convolution with the intrinsic noise only distribution separately for the repressed and the activated mode, each with its own best-fit variance of the shift distribution (here assumed to be Gaussian). The total probability of each mode is calculated from the ratio of durations the system resides within the distribution around the corresponding mode based on the empirical distributions (SDE simulation), assuming steady-state and ergodicity of the stochastic system with extrinsic noise. These total probabilities are then used to combine the two modes. The resemblance between the E2F distribution generated by the SDE model (green) and that by the extrinsic noise-convolved MGS (red) is evident in Figure 7C for two different levels of extrinsic noise (as defined for the SDEs in [51]).

## Discussion

Two key challenges in stochastic modeling of cellular networks are computational efficiency in describing intrinsic noise and adequate description of extrinsic noise. This study provides a modular approach that makes such computations more tractable. To compute intrinsic variability, a range of approaches for predicting intrinsic noise, ranging from modified Gibbs sampling to scaled CME solution to direct CME solution in order of increasing accuracy and decreasing efficiency, is presented. Our methods provide an efficient alternative to previous time-stepping and analytical methods for modeling noise in cellular networks. These techniques can implement a model quite accurately for certain systems. However, the time-stepping method can require great computational cost, especially in its most accurate form (the Gillespie algorithm), and does not necessarily provide an accurate representation of extrinsic noise. The direct analytical approach is desirable because it accounts exactly for intrinsic noise, but it is only feasible for the simplest biological networks. In principle, our approximate methods are generally applicable to cellular networks with arbitrary complexity.

Likewise, representation of extrinsic noise by convolution provides significant advantages both in its intuitive relationship to the final distribution and in its computational tractability (e.g. small number of parameters). Because extrinsic noise is a heterogeneous phenomenon with multiple sources, it is likely to have some components best modeled as variation in parameters and others best modeled in other ways. Applicability of each method can be evaluated by its ability to produce similar distributions to other methods, and more generally to account for different sources of noise. The convolution method, with its ability to mimic results from parameter perturbation methods, is useful in this regard. Also, we expect the convolution method to be highly flexible. Though it is developed in the context of analyzing steady-state distributions, it may also be applied to incorporate contributions of extrinsic noise into time-course simulations of stochastic network dynamics, for example by the Gillespie algorithm.

The convolution method is also not constrained to the Gaussian form of the extrinsic noise distribution used here: if appropriate in a system, different extrinsic noise distributions with different numbers of parameters could be used. The Gaussian was used here because it was likely the best distribution with a manageably low number of parameters in these cases, but other distributions, such as mixture models, could provide more realistic final distributions at the expense of larger parameter sets. In theory, this pattern could be continued to the point of using an unconstrained function as the extrinsic noise distribution to exactly fit the observed final distribution; while, as noted above, this would compromise the mechanistic insight from the analysis, it may be useful in characterizing the system in other ways, especially if the reaction parameters are known from other measurements.

Importantly, our study has defined a general, streamlined framework where one can derive unknown parameters from a distribution using fitting algorithms. For instance, our framework for extrinsic noise aids in obtaining initial estimates of reaction parameters based on the modes of the distribution, since these correspond well to the best-fit parameters. We have illustrated the basic concept of this approach through the analysis of two simple synthetic gene circuits as well as the feasibility of its application to a more complex cell cycle entry model. Due to the wide variety of perturbations that extrinsic noise can induce in all parameters and variables, however, we caution that apparent agreement with experiment could be seen for different models. To overcome this challenge, prior knowledge and alternative measurements are helpful for constraining the model, in terms of both reaction mechanisms and corresponding parameters. It is likely that this general framework is applicable for any biological network where a sufficiently mechanistic reaction mechanism is available. However, specific interpretations and applications of the fitted parameters, including those for the extrinsic noise distribution, will be context dependent. *Ad hoc* constraints and prior knowledge of the Markov chain describing the network dynamics, such as irreducibility, may sometimes be required, which usually demands no more mechanistic insights of the system than what's already required to carry out the actual sampling scheme.

## Methods

### Modeling a Toggle Switch

The following model is adapted from that of Gardner and colleagues [47]. Let the two proteins be U and V with molecule numbers *u* and *v* respectively. Based on Hill kinetics for synthesis and linear kinetics for degradation, the system can be described as:
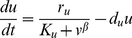
(20)

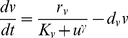
(21)A point in state space for this system is denoted (*u*, *v*). With appropriate parameters, the system can be bistable. In such a case, the deterministic steady states are at (*u_min_*,*v_max_*) and (*u_max_*,*v_min_*), where *u_min_*<*u_max_* and *v_min_*<*v_max_*. The number of states in this system that could potentially have nonnegligible probability is small enough that the CME at steady state can be solved analytically using linear algebra, provided the molecule numbers are small enough:
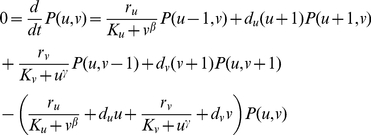
(22)Scaling allows solutions for larger amounts of protein.

The circuit can be induced by adding the antibiotic NFX.

Adding antibiotic to induce protein U's high state involves initiating an SOS response, which degrades protein V [48]; thus it brings about the perturbation
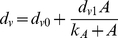
(23)where *d_v0_* is the basal degradation rate of V, *d_v0_*+*d_v1_* is its maximal degradation rate, *A* is the antibiotic concentration, and *k_A_* is the half-maximal constant for the enhanced degradation.

For experimental flow cytometry data, ON and OFF fractions of the data were determined by fitting the points to a mixture model consisting of two Gaussians using Mixmod 2.1.1. The theoretical data were partitioned based on which protein had a higher molecule number.

### Modeling a Growth-Modulating Positive-Feedback Circuit

Let *n* denote the number of T7 RNAP molecules in a given cell and let the cell have *M* promoters producing it, with *m* of them in an inactive state (O_0_) and *M-m* in an active state (O_1_). Tan *et al.* [49] modeled this system using the Gillespie algorithm with six reactions. Five are normal chemical reactions: synthesis of a T7 RNAP molecule from O_0_, with propensity *k_0_ m*, or from O_1_, with *k_1_m*; conversion of an O_0_ and a T7 RNAP molecule to an O_1_, with propensity *k_f_mn*, or the reverse, with *k_b_(M−m)*; and degradation of T7 RNAP, with propensity *d_x0_n*. The sixth is cell division, which distributes the T7 RNAP molecules according to a binomial distribution and resets all the promoters to O_0_, and has propensity
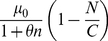
(24)Where *μ_0_* and *θ* are constants, *C* is the carrying capacity of the system, and *N* is the number of cells in it. When tracking molecule numbers in a single cell for purposes of determining steady state, one thus assumes, according to the binomial distribution, that cell division moves the molecule number from *n′* to *n* with probability
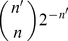
(25)For *n′*≥*n*.

It is useful to apply a somewhat different definition of steady state for this system than in more typical reaction systems. If all the reactions are required to reach steady state, then the system must be at carrying capacity, and thus cell division can be eliminated from the analysis; this results in a monostable circuit. However, provided that the intracellular reactions are fast on the timescale of the growth curve, temporary quasi-steady states at other points along the growth curve, for example at log phase, can exhibit significant additional properties, including bistability; thus steady-state analysis at these times can replicate the features observed by the Gillespie algorithm. To do this, let
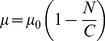
(26)be the effective growth rate, leading to the steady-state master equation
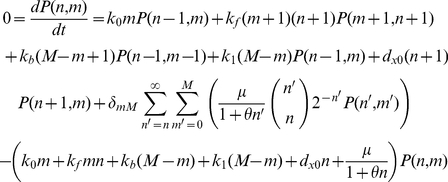
(27)


The network can be induced by IPTG, effectively increasing *k_0_* and *k_1_*. Different steady states can be investigated by examining the system at different OD levels; in each case *N*/*C* is estimated as the ratio of the current OD to the carrying-capacity OD.

ON/OFF fractions for this network were found by identifying the two most prominent peaks in the histogram of the protein being monitored and then defining the bin in between those peaks with the lowest value as the border between ON and OFF. When peaks were found to blur together (a problem in the theoretical distributions), the point on a shoulder with the lowest derivative (approximated as a finite difference between points) was designated as the border between the main peak and the shoulder “peak.”

### Characterization of the Toggle Switch Circuit

*E. coli*, JM2.300 was transformed with two plasmids, pTSMa and pCIRa [48]. The cells were cultured overnight at 37°C with 2 mM IPTG to ensure OFF state. The cells were then washed twice with fresh media, diluted 1000 fold, and cultured at 37°C. After three hours, cells were treated with various concentrations of NFX and further cultured at 37°C for 5 hours. Samples were then collected and subjected to flow cytometry analysis. LB medium was used throughout the experiment.

### Characterization of the T7 RNAP* Circuit

The construction and characterization of the T7 RNAP* positive feedback circuit were described by Tan *et al.* [49]. MC4100z1 cells (from Michael Elowitz) were used throughout the study.

### Modeling Myc/Rb/E2F Network

As an example to evaluate the feasibility of extending the proposed computational framework to a more complex model, we adopt a previously developed stochastic model for this network [51]. It consists of a set of stochastic differential equations, which has the general form of
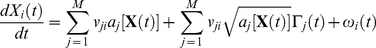
(28)where *X_i_*(*t*) represents the number of molecules of a molecular species *i* (*i = 1, …, N*) at time *t*, and **X**(*t*) = (*X_1_*(*t*), .., *X_N_*(*t*)) is the state of the entire system at time *t*. **X**(*t*) evolves over time at the rate of *a_j_*[**X**(*t*)] (*j = 1, …, M*), and the corresponding changes in the number of individual molecules are described in *v_ji_*, Γ*_j_*(*t*) and ω_i_(*t*) are temporally uncorrelated, statistically independent Gaussian noises. Γ*_j_*(*t*) is the standard normal distribution with mean 0 and variance 1. ω_i_(*t*) tunes the level of empirical additive extrinsic noise [51].

When ω_i_(*t*) is set to 0, the SDE simulation gives an approximation to the exact solution of the discrete stochastic chemical reaction system [52], against which the MGS distributions are compared. The inclusion of either low or high levels of extrinsic noise is realized by setting ω_i_(*t*) to 15 or 50, respectively. Based on the reactions involved in this system [51], we can write down the following CME:
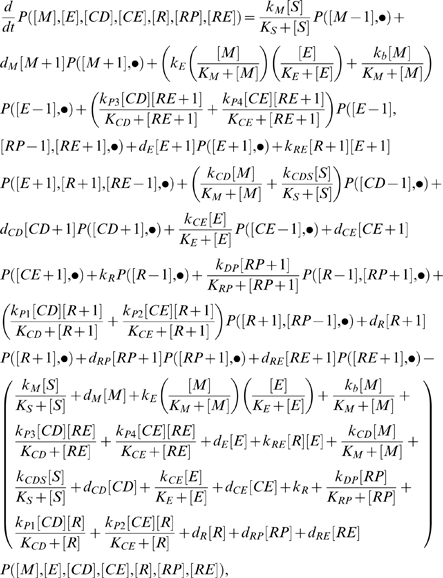
(29)where • represents the state of interest for the molecular species not specified, as in ([*M*], [*E*], [*CD*], [*CE*], [*R*], [*RP*], [*RE*]), which represent molecular number. Refer to Lee et al [51] for detailed description of the reaction mechanism and the corresponding rate constants.
